# Supplementary material for: Maize Transcription Factor ZmHsf28 Positively Regulates Plant Drought Tolerance
Source: Int J Mol Sci. 2023 Apr 29;24(9):8079. doi: 10.3390/ijms24098079 (PMC10179534; doi:10.3390/ijms24098079)
Supplement: Supplementary file 1 [file ijms-24-08079-s001.zip › Supplementary Figures and Tables.pdf]

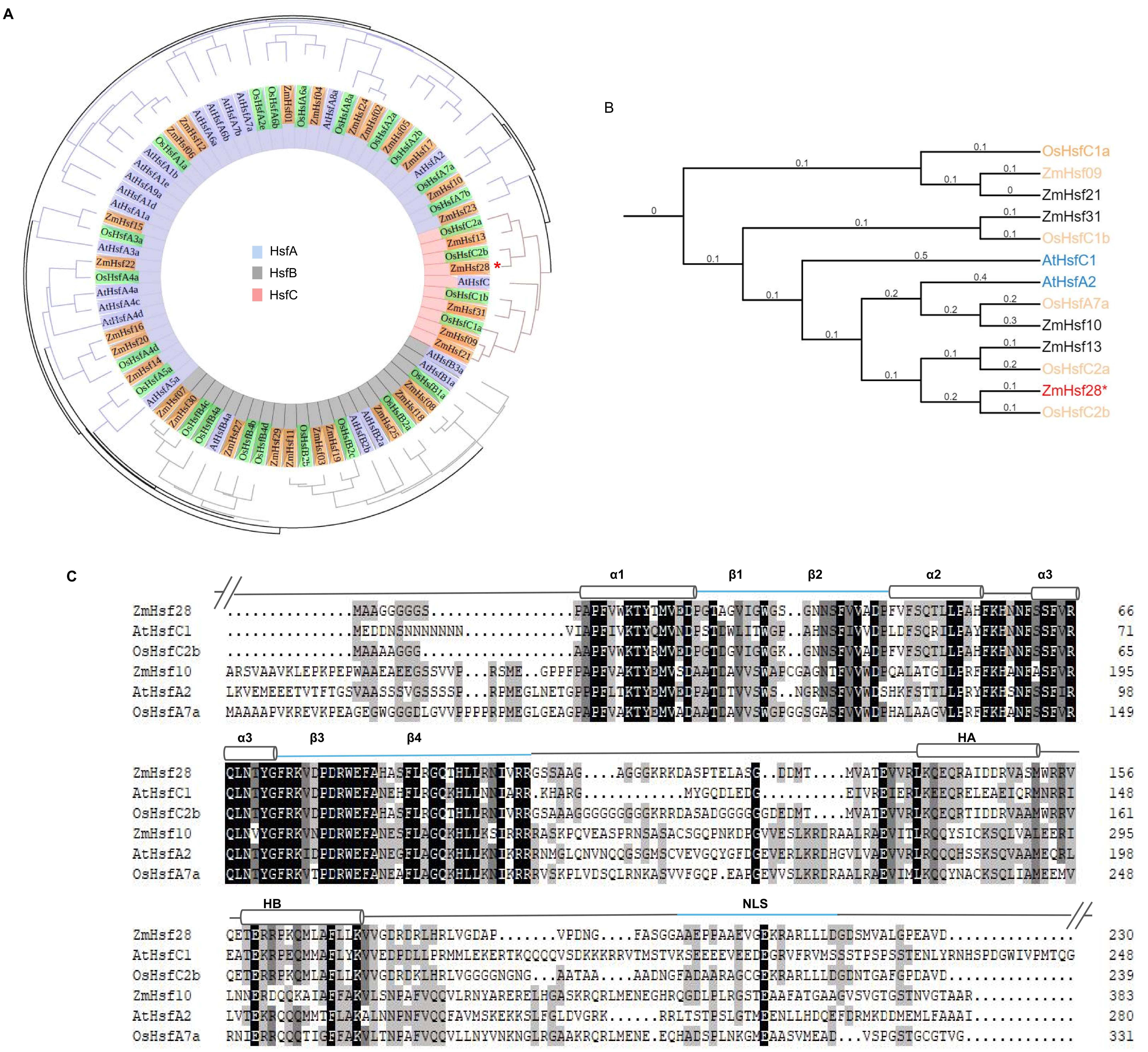

Figure S1. Phylogenetic analysis of ZmHsf28. (**A**) Phylogenetic trees of Hsfs from maize, rice and Arabidopsis. The A, B and C sub-families of Hsfs are indicated in different colors. ZmHsf28 is indicated by the asterik. (**B**) Phylogenetic analysis of ZmHsf28 with Hsfs in maize, rice and Arabidopsis. (**C**) Conserved domain analysis of ZmHsf28. The conserved DBD domain (α1-α3, β1-β4) and OD region (HA, HB) and nuclear location signal (NLS) are labeled.

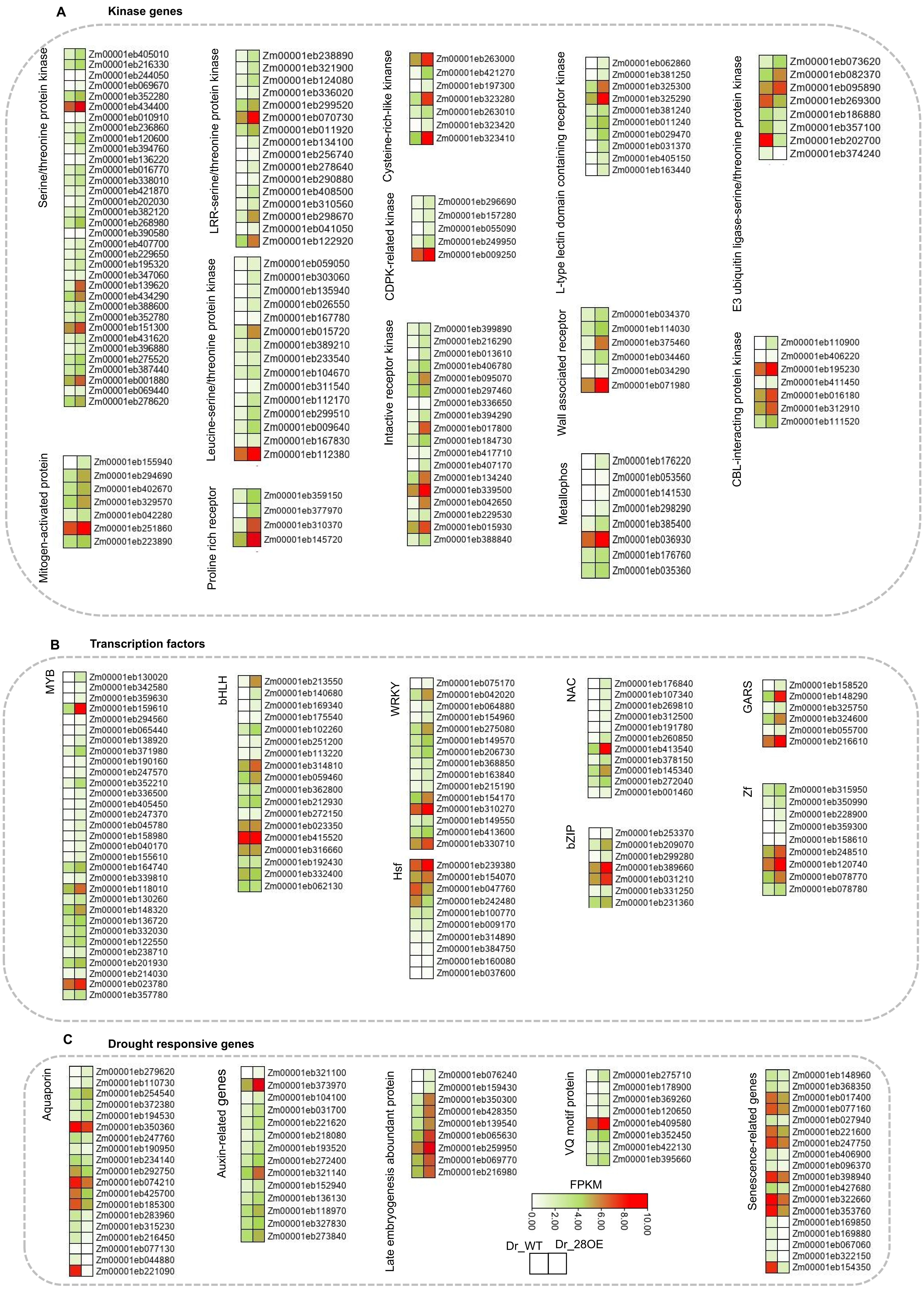

Figure S2. DEGs in ZmHsf28OE lines and WT maize plants under drought treatment. Heat maps of DEGs in ZmHsf28OE-1 maize lines compared to WT in response to drought including kinase genes (**A**), transcription factors (**B**) and other drought resposive genes (**C**). The colors indicate FPKM values.

Table S1. *cis*-element predication in gene promoters.

Genes	*cis*-elements	Position
ZmRD21	HSE	-1754，-1564，-1457，-1090，-1066，-258，-215
ZmCAT1	HSE	-1948，-1290，-1107，-1069，-511，-405
ZmZEP1	HSE	-935，-932，-371，-359，-135
ZmAAO4	HSE	-1732，-1561，-1457，-1422，-1270，-1170，-286，-191
ZmAOS1	HSE	-1881，-1540，-1401，-773，-461

Notes: The specific HSEs used for EMSA were labeled in red.

Table S2. Primers used for qRT-PCR analysis.

Genes	Accession Numbers	Left Primer (5'-3')	Right Primer (5'-3')
ZmHsf28	GRMZM2G118047	CCCTTCGTCTTCTCGCAGA	GGAAGCCATAGGTGTTGAGC
ZmNCED9	GRMZM5G838285	ACTTCTACTTCAGGCCCGAC	GACGTCTTCTCCCTGTCCAG
ZmAAO4	GRMZM2G141473	GATGATATCCCCGCTCCCAA	AAGTGCTTCGTCTCCCATCA
ZmZEP1	GRMZM2G379053	TTGATGTTCGGGAGCCTGAT	GCAGTAACCTTGAAGCCTGG
ZmRD21	GRMZM2G166281	CCATGACTACCCCATCTGCA	GCTCTTCATTCCGTCAGCTG
ZmRAB18	GRMZM2G098750	GGGTGTGGTGGAGAAGATCA	TTCTCGTGAGTGCCTTCAGT
ZmDREB2A	GRMZM2G006745	GTGCTGTGGTGCATGGT	CGTAGGCCCATCTCGTGATC
ZmPOD3	GRMZM2G126261	CTACAAGAACCTGCTGTCGC	GAGGTTCCCCATCTTCACCA
ZmLOX8	GRMZM2G104843	AGATCCAGGAGAACAGCGAG	GATCATGGACGGAGGAGGAG
ZmAOS1	GRMZM2G076225	CTACTACTTCCAGGGGCAGG	TCCACCTTGTCCATGTCGAA
ZmAOC1	GRMZM2G077316	CAACAAGGTGTACAACGGCA	AGAAGCTGTAGATGGCCTCG
ZmSOD4	GRMZM2G025992	AATGTGACAGCGGGAGAAGA	AAGCTCATGTCCACCTTTGC
ZmCAT1	GRMZM2G088212	GTGAATGCACCAAAATGTGC	TGATGCACTTCTCACGACAG

Table S3. Primers used for gene promoters cloning

Genes	Left Primer (5'-3')	Right Primer (5'-3')
ZmRD21	ATCACGTAGATAAACCCGCGTC	CTCTGCCGCGCTACAGGAA
ZmAOS1	CCTATCGATGTGGAGGATGTGTGGT	AGAGGAGGCTCGCAACAAGTTGCTT
ZmZEP1	AGCTGAGGATCTCCAATCACACGAC	ATCACCGAATTATATATCGATCGCG
ZmAAO4	CGGTGCGTTGTCTAATCAACACT	CTCGATCTCTCCTCTCCAACCTTCT
ZmCAT1	CAATCGGTGCCAACATGATGTGATA	TAGCACATAGGGCAAAATCGATGAA

Table S4. Probes used for EMSA analysis.

Promoters	Left Primer `Biotin (5'-3')	Right Primer (5'-3')
pZmAAO4	TCTTAATCTCCTAGAAAAATACGGT	ACCGTATTTTTCTAGGAGATTAAGA
pZmRD21	TATCGGTTTTTTTAGAAGATATTATGGC	GCCATAATATCTTCTAAAAAAACCGATA
pZmCAT1	GAAGATGAGCTTCTAGGACTGTTAGA	TCTAACAGTCCTAGAAGCTCATCTTC
pZmAOS1	GTCAAGTCGGGTCTAGAAACTCATAGGCA	TGCCTATGAGTTTCTAGACCCGACTTGAC
pZmZEP1	GGGTGTTTTTCTGGAATCATGCA	TGCATGATTCCAGAAAAACACCC

Table S5. Hsfs used for phylogenetic analysis.

HsfA					
**Genes**	**ID**	**Genes**	**ID**	**Genes**	**ID**
ZmHsf01	Zm00001d027757	*AtHsfA1a*	AT4G17750	*OsHsfA2a*	Os03858160
ZmHsf02	Zm00001d028269	*AtHsfA1b*	AT5G16820	*OsHsfA2b*	Os07g08140
ZmHsf04	Zm00001d032923	*AtHsfA1d*	AT1G32330	*OsHsfA2e*	Os03853340
ZmHsf05	Zm00001d034433	*AtHsfA1e*	AT3G02990	*OsHsfA3a*	Os02g32590
ZmHsf06	Zm00001d034886	*AtHsfA2*	AT2G26150	*OsHsfA4a*	Os01g54550
ZmHsf10	Zm00001d044259	*AtHsfA3a*	AT5G03720	*OsHsfA4d*	Os05g45410
ZmHsf12	Zm00001d012823	*AtHsfA4a*	AT4G18880	*OsHsfA5a*	Os02g29340
ZmHsf14	Zm00001d016520	*AtHsfA4c*	AT5G45710	*OsHsfA6a*	Os10g28340
ZmHsf15	Zm00001d016674	*AtHsfA5a*	AT4G13980	*OsHsfA6b*	Os03g06630
ZmHsf16	Zm00001d038746	*AtHsfA6a*	AT5G43840	*OsHsfA7a*	Os01g39020
ZmHsf17	Zm00001d018941	*AtHsfA6b*	AT3G22830	*OsHsfA7b*	Os06836930
ZmHsf20	Zm00001d010812	*AtHsfA7a*	AT3G51910	*OsHsfA8a*	Os03812370
ZmHsf22	Zm00001d012749	*AtHsfA7b*	AT3G63350	*OsHsfA1a*	Os03863750
ZmHsf23	Zm00001d046204	*AtHsfA8a*	AT1G67970		
ZmHsf24	Zm00001d048041	*AtHsfA9a*	AT5G54070		
ZmHsf26	Zm00001d033987				
**HsfB**					
**Genes**	**ID**	**Genes**	**ID**	**Genes**	**ID**
ZmHsf03	Zm00001d031736	*AtHsfB1a*	AT4G36990	*OsHsfB1a*	Os09g28354
ZmHsf07	Zm00001d005843	*AtHsfB2a*	AT5G62020	*OsHsfB2a*	Os04g48030
ZmHsf08	Zm00001d005888	*AtHsfB2b*	AT4G11660	*OsHsfB2b*	Os08g43334
ZmHsf11	Zm00001d052738	*AtHsfB3a*	AT2G41690	*OsHsfB2c*	Os09g35790
ZmHsf18	Zm00001d020714	*AtHsfB4a*	AT1G46264	*OsHsfB4a*	Os08g36700
ZmHsf19	Zm00001d021263			*OsHsfB4b*	Os07844690
ZmHsf25	Zm00001d026094			*OsHsfB4c*	Os09g28200
ZmHsf27	Zm00001d029270			*OsHsfB4d*	Os03825120
ZmHsf29	Zm00001d022295				
ZmHsf30	Zm00001d020704				
**HsfC**					
**Genes**	**ID**	**Genes**	**ID**	**Genes**	**ID**
ZmHsf09	Zm00001d044168	*AtHsfC*	AT3G24520	*OsHsfC1a*	Os01843590
ZmHsf13	Zm00001d016255			*OsHsfC1b*	Os01853220
ZmHsf21	Zm00001d011406			*OsHsfC2a*	Os02g13800
ZmHsf28	Zm00001d046299			*OsHsfC2b*	Os06g35960
ZmHsf31	Zm00001d043536				
